# Assessment of Impacts of Coal Mining in the Region of Sydney, Australia on the Aquatic Environment Using Macroinvertebrates and Chlorophyll as Indicators

**DOI:** 10.3390/ijerph15071556

**Published:** 2018-07-23

**Authors:** Aal-e Ali, Daniel R. Sloane, Vladimir Strezov

**Affiliations:** Department of Environmental Sciences, Faculty of Science and Engineering, Macquarie University, Sydney, NSW 2109, Australia; aal-e.ali@students.mq.edu.au (A.-e.A.); daniel.sloane@hdr.mq.edu.au (D.R.S.)

**Keywords:** aquatic life, environmental impairment, coal mine, ecosystem, macroinvertebrate, industrial pollution, Stream Invertebrate Grade Number-Average Level (SIGNAL)

## Abstract

Coal and coal seam gas mining have impacts on the water and sediment quality in the proximity of the mining areas, increasing the concentrations of heavy metals downstream of the mine discharge points. The objective of this study was to assess the impact of coal mining on the environment in the Sydney region, by investigating macroinvertebrates and chlorophyll as indicators of industrial pollution and environmental impairment. The study revealed changes in abundance, taxonomic richness, and pollution sensitive macroinvertebrate groups. A statistical evaluation of the aquatic life was performed and a correlation of the contaminants with the presence of a community in the ecosystem were studied. The environmental sustainability of the investigated rivers and streams with water chemistry affecting the biological system was assessed. A non-uniformity in the changes were observed, indicating a difference in the tolerance level of different invertebrates.

## 1. Introduction

Rapid development with growing energy consumption has resulted in an exponential production of resources for energy generation, such as coal and natural gas. During mining, a variety of materials are consumed, processed, and washed away as a discharge, in the form of the produced water. Although the water that is discharged into the creeks and waterways is diluted in the downstream flow, a continuation in the discharge leads to a persistence of contaminants in the waterways. Water and sediment quality can be compromised because of coal and coal seam gas mining activities [1,2]. During the production, coal washing and groundwater seepage into underground mines generate contaminated wastewater. Sulphur containing material in mine comes in contact with water, which naturally generates sulphuric acid, termed as acid mine drainage (AMD), which can dissolve harmful metals and metalloids from the surrounding rocks and increase the water contamination level [3]. Alkaline dozers are used to reduce the acidity and dissolved metals from the acid mine drainage, but the improvements were shown as non-linear [4].

The wastewater discharged from coal mines in the water streams can compromise water quality, geochemistry, and metal contamination [1], causing a long distance impact on the ecosystem [5]. Different heavy metals discharged by coal mines can be consumed by the living aquatic organisms [6]. The aquatic ecological impact of mine wastewater is evident in the literature [7], however the correlation of the impact with multiple parameters, including water quality indices, sediment quality indices, and individual contaminants concentration, are still not available.

Low soluble trace metals can be easily adsorbed in sediments, and this continuous process yields to a high accumulation of contaminants [8]. Because of sediments being the ultimate sinks for trace metals, the living organisms on the coastal areas may also be affected by the poor sediment quality. Certain trace metals, which are not permanently bonded in the sediment, can be released in the water under different conditions [9]. Some of them (Mo, Cr, Zn, Se, Co, and Fe) are used in the biological functions and are not harmful in low concentrations, but those that are not essential for the biological use (Pb, Hg, Cd, As, and Ni) can be toxic at very low concentrations [10]. The trace metals can be accumulated in the body of the biota and finally end up in the food chain and affect human health [11].

The increment of trace metals in the discharged water was observed previously in the downstream sediment and surface water from the coal mining activities in the Sydney region [1]. There are certain species that have their own system to maintain the intake of contaminants and there is constant accumulation of trace metals in these species, irrespective of the available trace metals in the water system [12]. The increase in salinity due to discharge may affect the inhabitants in different ways. In some cases, the salinity increment can be tolerated by the inhabitants [13], on the other hand, algae and duckweed are affected by the saline water discharge from the mines [14]. The effect of the salinity increment on invertebrates can lead to high densities (i.e., abundance per square area of stone and low richness) [15]. There are acidophilic bacteria that cause coal to leach and form low pH drainage by their metabolic activity [16]. The inhabitants have their characteristic affinity towards the available trace nutrients. In presence of certain trace metals, the process of the uptake of other trace metals by inhabitants is reduced. Even these processes, including photosynthesis, are affected [17]. The selective nutrient uptake of trace metals affects in both ways by increasing or decreasing the damage to the inhabitants. Eventually, the bioavailability of the trace metals affecting the inhabitants are reflected in diverse forms. It is reported that aquatic invertebrates consume and accumulate trace metals in varying concentrations across taxa.

Because of the variety of behavioral changes, macroinvertebrates in a river ecosystem can be used as a tool for the assessment of the water quality and industrial impact [18]. They can be applied as anthropogenic adverse effect indicators on aquatic systems [19]. The impact of wastewater discharge on water, sediment, and damage to aquatic ecosystems may have some correlation. The aim of this work is to investigate the changes in the ecology of rivers by the analysis of chlorophyll and invertebrates in the area affected by the produced industrial discharge from coal mining activities, and in the less affected areas before the discharge points.

## 2. Materials and Methods

### 2.1. Study Area and Site Description

**Figure 1:**
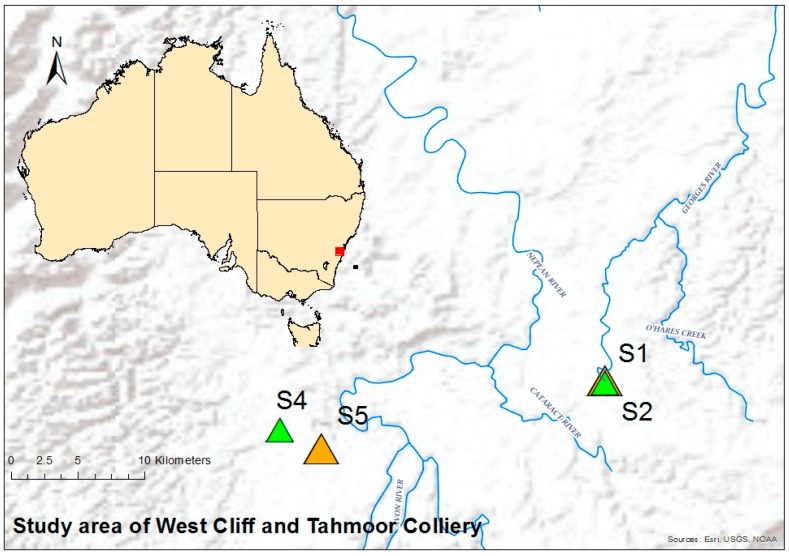
Sampling locations in south Sydney area for West Cliff and Tahmoor Colliery.

The selected study site in this work was located 60 to 120 km southwest of Sydney, Australia. Two mines in the region were selected for this study, one was the West Cliff Colliery, which discharges into Brennans Creek and then to Georges River, and the other was the Tahmoor Coal Mine, discharging into the Bargo River. The coordinates and details of the sampling locations are listed and illustrated in Table 1 and Figure 1, respectively. The samples were collected from the locations before (upstream) and after (downstream) the produced water discharge points. The sampling points were selected in the same way as in the previous study [1], because the water quality in the upstream and downstream of the mine discharge was investigated, and the indices were already calculated and reported for the assessment of the water quality.

### 2.2. Sampling and Analysis

The aquatic macroinvertebrate samples were collected from both the upstream and downstream locations to the coal mine waste discharge points. The study was carried out in October 2016. Two types of samples were collected for this study, (i) stone scratched samples for chlorophyll-A analysis, and (ii) stone scrubbed samples for benthic macroinvertebrate analysis. A standard 18” rectangular frame net with a mesh size of 500 µm was used for sampling of the invertebrates. Six randomly selected benthic macroinvertebrate samples were collected from the coble riffle available in the area within a 10-m distance of each site, using a ‘kick sampling’ method. In this method, the sampling net was placed at the bottom and the stream was disturbed before the net, to let the invertebrates come out of their colony and get trapped in the net. The samples collected were transferred to small jars and stored in ice cooler.

The second technique of sample collection for the study was taken by the scrubbing and scratching of the stone to examine the biota on a rock for the abundance and type of biota. Invertebrates and chlorophyll-A were both taken from randomly selected rocks, which were removed from the river at sites above (upstream) and below (downstream) the mine water discharge. The rocks of a football-like size were removed from the stream bed and placed into a 500 μm mesh net while still under water. The net was placed immediately next to the rock to capture any animals dislodged during the rock removal. The rocks were taken to the river bank, and the rock and the contents of the net were placed into a plastic tray for processing. A 3 cm by 3 cm template was used twice for the collection of the chlorophyll from a rock sample. The template was placed on the rock, the algae and biofilm of entire area of rock were carefully scrapped within the template and rinse transferred to a jar using a wash bottle. The jar containing the algal scraping was wrapped in aluminium foil to avoid light exposure and placed in a portable cooler. For the chlorophyll-A analysis, the samples were filtered first through a Whatman 42 membrane filter. The filter membranes were kept in acetone at 4 °C for 24 h in the dark. The acetone solution was then analysed for chlorophyll-A by measuring the absorbance at 750, 664, 647, and 630 nm, using a Hach portable spectrophotometer. The calculation of the chlorophyll concentration was performed according to the standard procedure, described elsewhere [20].

The sampling to examine the biota on a rock for the abundance and type of biota was performed on the same stone where the chlorophyll samples were scratched from. The rocks were thoroughly scrubbed and washed in the tray, along with the net. It was visually inspected to ensure that all of the animals were removed. All of the animals were transferred into a clean plastic jar and frozen until the later analysis in the laboratory. The abundance and type of invertebrates from the rock were examined, sorted, identified, and enumerated under a high-resolution microscope, following the guidelines of Environmental Protection Agency [21]. The invertebrates were identified to the family level, where possible, apart from Chironominae, which were identified to the subfamily. The oligochaetes and mites were identified to their class only. The invertebrates collected were analysed for their abundance, density, and richness. The abundance was described as the number of animals collected on each rock, and the density was calculated as number of animals collected on each rock, divided by the size of the rock. The richness was the number of different invertebrate taxa collected on each rock.

The surface area of each rock was estimated by covering the rock in a single layer of aluminium foil and calculating the area from the mass of the foil, based on a standard curve. The abundance, richness of invertebrates, densities of invertebrates, and chlorophyll-A on the rocks collected before (upstream) and after (downstream) the mine wastewater discharge points were compared using a one-way analysis of variance (ANOVA). The assumptions of normality and the homogeneity of variance were determined using q–q plots and plots of the residuals. The significance level (α) for these analyses was 0.05. The analyses were done using Minitab v17 (Minitab Pty Ltd., State College, PA, USA), IBM SPSS Statistics 24 (SPSS Inc, Chicago, IL, USA), and Past 3 software (Øyvind Hammer, Natural History Museum, University of Oslo, Norway).

### 2.3. Stream Invertebrate Grade Number-Average Level (SIGNAL)

Stream Invertebrate Grade Number-Average Level (SIGNAL) is macroinvertebrate scoring system for samples from the Australian River. It can indicate the pollution type and the factors affecting the invertebrate community, which helps in deciding the condition of the river or the river health. A higher SIGNAL-2 score is related to higher water quality, indicating low salinity, turbidity, nutrient (e.g., nitrogen and phosphorus) contents, and a high content of dissolved oxygen [22]. The SIGNAL-2 score was calculated by identifying the invertebrates to the family level. Each type of invertebrate is assigned a grade number between 1 and 10, and their abundance has weight factors accordingly. The SIGNAL score is calculated as follows:

$$\mathrm{SIGNAL}\;\mathrm{score}=\frac{\mathrm{Total}\;\mathrm{of}\;\mathrm{grade}\;\times \mathrm{weight}\;\mathrm{factor}}{\mathrm{Total}\;\mathrm{of}\;\mathrm{weight}\;\mathrm{factor}}$$

### 2.4. Statistical Analysis ANOVA

This is a simple test of analyzing the mean and variance among and between the groups. This could be one-way ANOVA or two-way ANOVA, depending on the number of variables compared to the other variables. The analysis of abundance of the invertebrates for its significance was performed on a one-way ANOVA using Minitab v17, IBM SPSS Statistics 24, and Past 3 statistical software. The quality data were compared between the sites and stream levels, to analyze the normality and homogeneity of variance in order to find its significance.

### 2.5. Simpson’s Dominance Index (D)

The formation of a continuous progression from the dominants through the intermediates, to the rare species, by arranging the communities in a sequence from most to least important, generates a dominance index. It directs towards the submissive and aggressive behavior between two members of all of the possible paired combinations of animals in the group. This index takes species richness and the abundance of individuals within each species into account, in order to determine how uneven a biological community is. This index is calculated from pooled samples at each site to yield a value between 0 and 1. This value represents the probability that two randomly selected individuals will be from the same species. Therefore, a probability of 1 means that all of the individuals at a site belong to the same species, and a value of 0 indicates that the individuals are evenly distributed amongst all of the species from a community. The following equation calculates this index [23]:

$$D=\underset{i}{\sum }{\left(\frac{n_{i}}{n}\right)}^{2}$$

where *n_i_* is the number of individuals of taxon *i*.

### 2.6. Similarity Indices

Numerous similarity indices are used to measure the similarity among the communities. Two suitable indices were considered for this study, Jaccard’s similarity index and Bray-Curtis dissimilarity index.

Jaccard’s similarity index is used to assess the similarity between biological communities, based on the presence and absence of taxa. In this comparison of two sets (communities), the intersection (taxa common to both communities) is divided by the union (total combined taxa from both communities) to yield the similarity index. The following equation represents this:

$$J\left(A,B\right)=\frac{\left|A\cap B\right|}{\left|A\cup B\right|}=\frac{\left|A\cap B\right|}{\left|A\right|+\left|B\right|-\left|A\cap B\right|}$$

where A and B are two sets of communities [24,25].

The Bray-Curtis dissimilarity index differs from Jaccard’s similarity index. In addition to presence and absence, Bray-Curtis takes the abundance of individuals within taxonomic groups into account. The Bray-Curtis similarity returns a value between 0 and 1, where 1 means that two sets do not share any similarity with each other at all, and 0 means that two sets have the same composition. It is represented as follows:

$$BC_{ij}=1-\frac{2C_{ij}}{S_{i}+S_{j}}$$

where *S_i_* and *S_j_* are the number of specimen counted for set *i* and set *j*, respectively, and *C_ij_* is the sum of the specimen that are common in both sets of the species [24,26].

## 3. Results and Discussion

**Figure 2:**
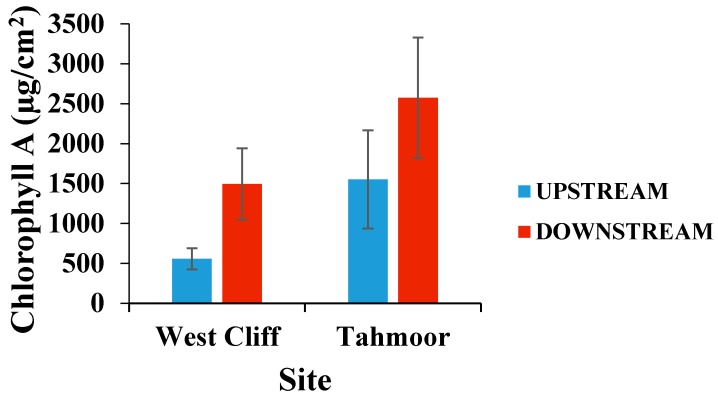
Mean (± standard error [SE]) chlorophyll-A densities on rocks collected from upstream and downstream of the mine water discharge points at Georges River (West Cliff Mine) and Bargo River (Tahmoor Mine). *n* = 6.

Figure 2 displays the mean chlorophyll-A densities on rocks collected from upstream and downstream of the mine water discharge points. At both sites, the density of chlorophyll on the rocks was not statistically significant (*p* > 0.05), however, it was observed that the chlorophyll abundance on the rocks was higher in the areas downstream of the mine discharge points. A statistical evaluation of chlorophyll-A revealed that there was a significant change in the chlorophyll-A density with the change in stream type (*p* = 0.028, F = 510.7, and df = 1, 1). Chlorophyll-A also showed a significant change with the change in site location (*p* = 0.026, F = 580.5, and df = 1, 1), which means that the change in the chlorophyll-A content in West Cliff was significantly different from the chlorophyll-A content of Tahmoor. The chlorophyll-A content in Waste Cliff was 558 µg/m^2^, while in Tahmoor it was 1551 µg/m^2^, indicating the environmental variability of the chlorophyll-A content even prior to the discharge point.

However, the difference in chlorophyll-A between the upstream and downstream locations at West Cliff was more than 2.6 times, while in Tahmoor it was more than 1.6 times, indicating that the downstream environment was more suitable for chlorophyll-A at both sites.

**Figure 3:**
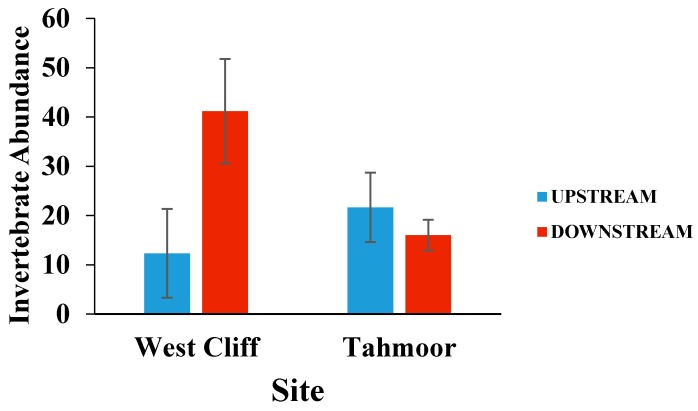
Mean (± SE) invertebrate abundance on rocks collected from upstream and downstream of mine water discharge points at Georges River (West Cliff Mine) and Bargo River (Tahmoor Mine). *p* = 0.015.

Figure 3 shows the mean invertebrate abundance on the rocks collected from upstream and downstream of the mine water discharge points. There was significantly greater invertebrate abundance on the rocks downstream of the West Cliff mine discharge point compared to the upstream sampling location (*p* = 0.015). The greater abundance of invertebrates downstream of the Westcliff mine discharge may reflect the habitat rather than the water quality difference, as a result of the mine discharge [27]. The flow velocities and water volume were both greater at the downstream than the upstream sites. There was no significant upstream to downstream difference in the invertebrate abundance at Tahmoor, however, a small decrease in the invertebrate abundance was observed in the downstream location.

**Figure 4:**
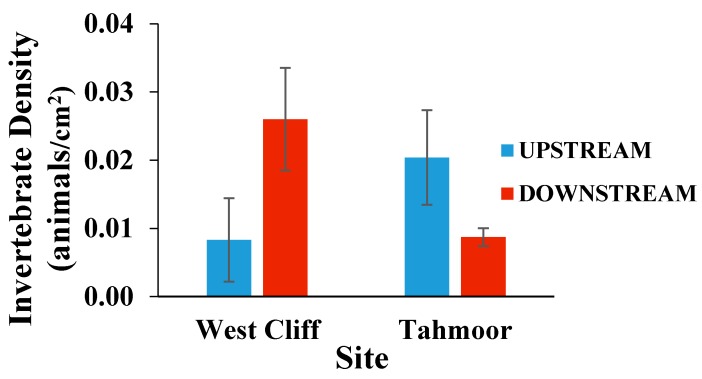
Mean (± SE) density of invertebrates on rocks collected from upstream and downstream of the mine water discharge points at Georges River (West Cliff Mine) and Bargo River (Tahmoor Mine).

The density of the invertebrates, shown in Figure 4, was evaluated by the abundance per unit area of the rock, and the results contrasted with each other on both sites. Although the results were statistically insignificant, they showed an increment in the downstream of the West Cliff site. On the other hand, the downstream of the Tahmoor site had a lower invertebrate density compared to the upstream sampling site. A decrease in the density of the invertebrates in the Tahmoor downstream can be considered as indicative of halo-sensitive invertebrates in the area [28]. There was no significant difference in the density (abundance/rock size) at either site.

**Figure 5:**
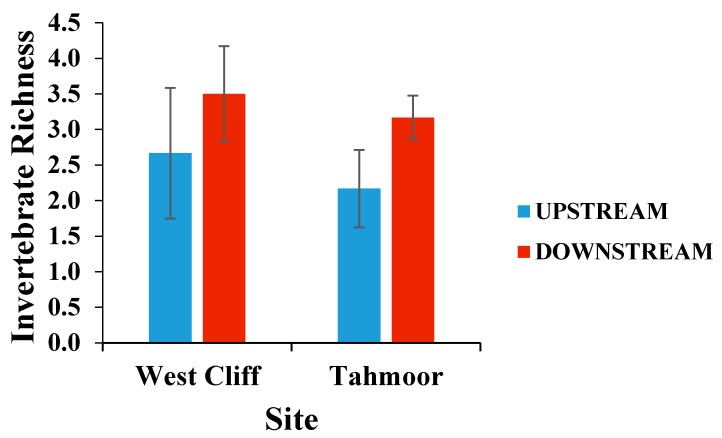
Mean (± SE) invertebrate richness per rock collected from the upstream and downstream of mine water discharge points at Georges River (West Cliff Mine) and Bargo River (Tahmoor Mine).

The invertebrate richness was not significantly different between the upstream and downstream locations in either of the rivers (*p* > 0.05), as presented in Figure 5, however, there was an apparent increase in richness in the downstream of both the West Cliff and Tahmoor sites, suggesting that the nutrient level is within the threshold limit of the contaminants for the survival of the species.

A scoring system of macroinvertebrate (Water Bug) in Australian rivers called Stream Invertebrate Grade Number–Average Level (SIGNAL), which indicates the water quality of the collected inhabitants, was calculated in this work. The SIGNAL-2 values were compared between the sites (West Cliff and Tahmoor) and between the stream locations (upstream and downstream), in order to elucidate the differences between these groups. The statistical assumptions for the valid application of the analysis of variance (ANOVA) include the normality and homogeneity of variances. These assumptions were tested with the Shapiro–Wilk test [29] and Levene’s test [30], respectively.

The Tahmoor site (TC) was found to violate the normality (0.632, df = 12, *p* ≤ 0.001), while the West Cliff site (WC) accepted the null hypothesis of normality (0.923, df = 12, *p* = 0.311). The null hypothesis of the homogeneity of variance by Levene’s test was met across the sites (0.358, df = 1, 22, *p* = 0.556). ANOVA is robust to the violations of normality, provided that the homogeneity of the variance assumption is met [31]. Therefore, the application of ANOVA was justified to compare sites, based on SIGNAL-2.

The upstream and downstream SIGNAL-2 values were then assessed to ascertain whether they met the assumptions of ANOVA. The downstream SIGNAL-2 accepted the null hypothesis of normality (0.927, df = 12, *p* = 0.347), while the upstream violated the normality (0.805, df = 12, *p* = 0.011). Once again, the null hypothesis of the homogeneity of variance by Levene’s test was met (0.087, df = 1, 22, *p* = 0.770), thus allowing the application of ANOVA, in order to compare the SIGNAL-2 between the stream locations.

**Figure 6:**
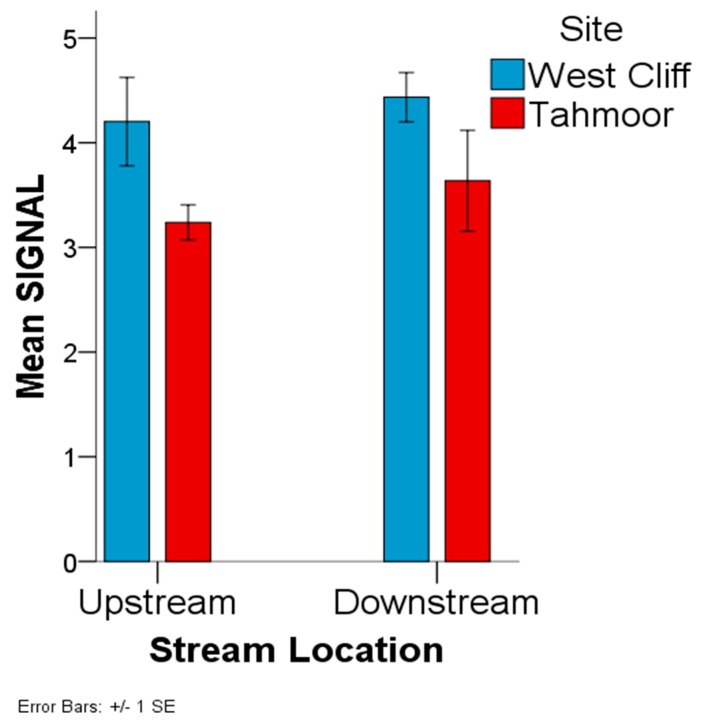
Stream Invertebrate Grade Number-Average Level (SIGNAL)-2 based on the invertebrates counted in the sample collected from the West Cliff Mine and Tahmoor Mine discharge area.

There was a statistically significant difference in the SIGNAL-2 scores between the West Cliff and Tahmoor sampling sites (F = 6.626, df = 1, 22, *p* = 0.017; Figure 6). West Cliff (M = 4.318, SD = 0.808) had a significantly higher mean tSIGNAL-2 score than the Tahmoor site (M = 3.438, SD = 0.868). Although the downstream locations displayed a higher mean SIGNAL-2 score (M = 4.036, SD = 0.979), than the upstream locations (M = 3.72, SD = 0.903) for both of the sampling sites, there was no statistically significant difference in the mean SIGNAL-2 score between the upstream and downstream locations (F = 0.675, df = 1, 22, *p* = 0.420; Figure 6).

**Figure 7:**
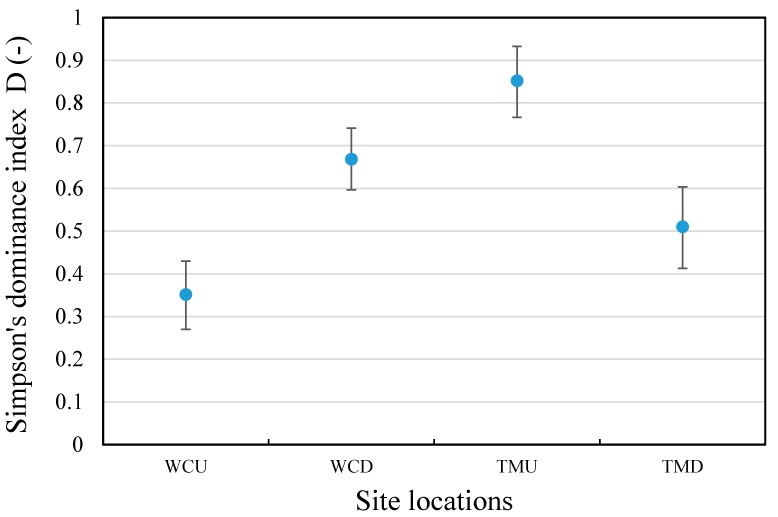
Simpson’s dominance index by site and stream location. WCU—West Cliff upstream; WCD—West Cliff downstream; TMU—Tahmoor upstream; TMD—Tahmoor downstream.

The macroinvertebrate dominance analysis of each site was carried out by pooling the subgroups of Chironominae (purple and lower) into Chironominae, and subgroups of Elmidae (Elminae Adult and Larvae) into Elmidae. The results are shown in Figure 7. The Tahmoor upstream location had the most uneven community, followed by the West Cliff downstream, Tahmoor downstream, and West Cliff upstream location, which had the most even community. There was no consistent effect of discharge on the macroinvertebrate dominance, as there was an increase in the dominance at the West Cliff downstream location, whilst a decrease at the Tahmoor downstream location.

**Figure 8:**
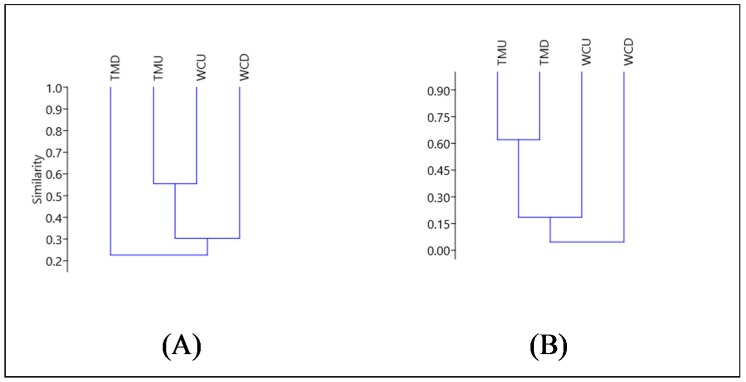
Jaccard’s similarity index (**A**), Cophenetic correlation coefficient = 0.9839; and Bray-Curtis dissimilarity index (**B**), Cophenetic correlation coefficient = 0.9894.

Jaccard’s similarity index, shown in Figure 8, was used to assess the similarity in the taxa present in the invertebrate communities at the sampling sites, both for the upstream and downstream of the mine discharge locations. It was determined that the present taxa of the invertebrate communities were more dependent on the location relative to the mine discharge points (upstream or downstream) than the sites (i.e., West Cliff or Tahmoor). Therefore, this study reveals that the mine discharge location is more relevant to the presence and absence of a specific invertebrate taxa than the location, for the studied sites. The upstream locations for both sites exhibited the closest similarity (Jaccard = 0.556), whilst the downstream locations were less similar from one another (Jaccard = 0.214).

The Bray-Curtis dissimilarity index, which takes presence/absence and abundance into account, displayed a different relationship to Jaccard. The Bray-Curtis is a dissimilarity index, where a value closer to 0 indicates a greater similarity between groups. Once the abundance of individuals within taxonomic groups was included in the dissimilarity algorithm, the stream locations continued to exert a greater influence on macroinvertebrate community composition than the site. This effect was particularly prevalent at Tahmoor, where the upstream and downstream locations were highly dissimilar (Bray-Curtis = 0.620), whereas the West Cliff upstream and downstream were more similar (Bray-Curtis = 0.106). Therefore, it appears that mine site pollution may be exerting a greater effect on the macroinvertebrate community composition at Tahmoor than at West Cliff, although a greater replication of the physiochemical sampling and macroinvertebrates would be required to confirm this.

**Figure 9:**
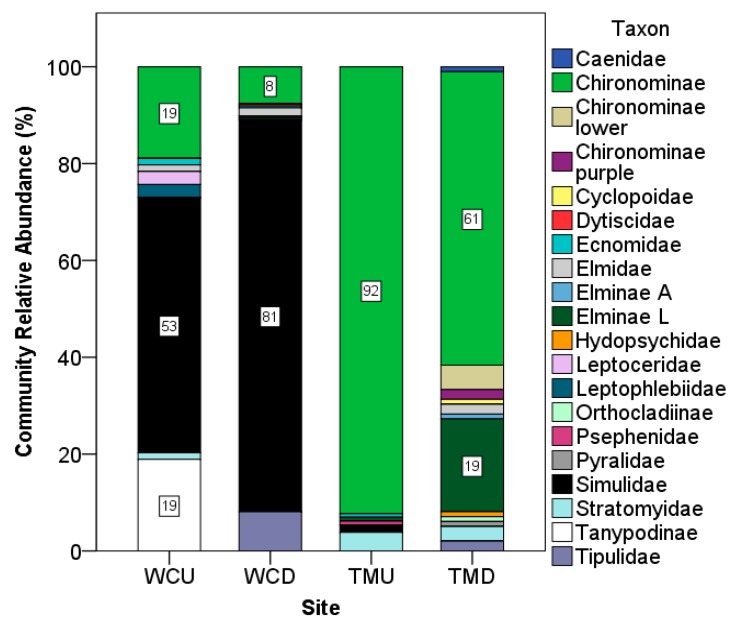
Relative abundance of invertebrate communities. WCU—West Cliff upstream; WCD—West Cliff downstream; TMU—Tahmoor upstream; TMD—Tahmoor downstream.

The percent of relative abundance of each taxon was calculated for each site and stream location, and the result is summarized in Figure 9. At West Cliff, the relative abundance of Simulidae increased at the downstream site (81%), compared with the upstream site (52.7%). Tipulidae, which was not present upstream, made up 8.9% of the downstream invertebrates. Conversely, Chironominae decreased from 18.92% upstream to 7.692% downstream. Tanypodinae, which was present at the same proportion as Chironominae upstream (18.92%), did not appear downstream. Leptophlebiidae and Leptoceridae were rare taxa upstream (2.7% each) and were not found downstream.

At Tahmoor, Chironominae made up most of the invertebrate community upstream (92.31%), although this taxon was found in a lower relative abundance downstream (60.61%). However, an increase in both sub-groupings of Chironominae (termed here as Chironominae lower and Chironominae purple), which were not found upstream, were present downstream at 5.05% and 2.02%, respectively. Similarly, a subgroup of Elminae (here termed Elminae L or Larvae), which were only found upstream at a 0.77% relative abundance, were found downstream at 19.19%.

If the West Cliff and Tahmoor upstream environments are taken to be representative indicators of the background macroinvertebrate communities at these sites, these are distinct even without the influence of mine site pollution. At the West Cliff upstream, the community is dominated by Simulidae, at a 52.7% relative abundance, increasing to 81% downstream, which suggests that this taxon is more tolerant to toxic conditions than other co-occurring taxa and/or that it had a greater capacity to adapt, perhaps due to a presumably larger gene pool to draw upon, which is inferred from its high relative abundance.

In contrast, Simulidae only made up 1.5% of the upstream community at Tahmoor, and were not found downstream. Instead, the Tahmoor upstream community was dominated by Chironominae at 92.3%. This declined substantially downstream, to 60.6%, although Chironominae remained the dominant taxon.

A correlation between the measurements conducted in this work with the water quality parameters studied in past for the same locations [1] is presented in Table 2. It was found that the richness of the invertebrates increases with pH, nitrate, dissolved solids, conductivity, and heavy metal potential index (HPI), indicating that the basic medium with nutrients favors the richness of invertebrates. However, it was found that the dissolved oxygen and the Environmental Water Quality Index (EWQI) were inversely related to the richness, indicating that a high presence of oxygen does not favor the richness. EWQI, an indicator of an adverse effect on living organisms, has clearly indicated the inverse trend in richness, SIGNAL, average sensitivity grade, and density of invertebrates. The water quality index also presents a reverse trend to the invertebrate richness, indicating that the bad water quality, to a certain degree, is favorable for the invertebrate’s life. This is also evident with the contamination index (C_d_), directly related to the invertebrate density and abundance. Based on this, it can be concluded that in good water quality, a low number of sensitive species are present, but in poor water quality, a high number of tolerant species of invertebrates are observed. This statement is more supported by the finding that the richness increment with pH is reversed in the case of the average sensitivity grade, additionally, the increased sensitivity average was in accordance with the water quality index, indicating that the more sensitive species are more populated in better water quality. An increment in the invertebrate density and chlorophyll content was directly related to the nitrate concentration [1], confirming the nutrient property of nitrate in the environment.

## 4. Conclusions

The comparative study of the chlorophyll and invertebrates in the upstream and downstream locations of two coal mines resulted in an inconsistent trend at different locations, which indicated that the chlorophyll and invertebrate do not behave identically to the contaminants in the environment for their survival. However, the nutrient content in the environment affects both of them in the same manner. The study observed that the discharge from the coal mines may alter the macroinvertebrate assemblages, as the most contaminant tolerant species were more prevalent in the downstream discharge locations. The invertebrate abundance relation with the contaminants was limited to the non-nutrient contaminants only. It was seen that a single parameter in the water quality could not be used to establish the trend in impact, as a variety of factors and concentration limits influence the trend. This behavior also suggested that the default guidelines may not necessarily be equally affecting the variety of taxa, and that other environmental factors may be impacting the abundance and distribution of the biota. Diverse assemblages have suggested pollution sensitive taxa and the essence of a sustainable development approach for the industrialization, by formulating measures to control the pollution sources.

## Figures and Tables

**Table 1:** Sample (S) and site identification.

S. No.	Mines/Industry	Sample Collection Site	Coordinates		Site I.D.
1	West Cliff Colliery Appin	Georges River, Appin, upstream	4°12′13.46″ S	150°47′52.74″ E	S1
		Georges River, Appin, downstream	4°12′17.25″ S	150°47′55.89″ E	S2
2	Tahmoor Coal Mine	Bargo River, Tahmoor, upstream	4°14′12.11″ S	150°34′46.02″ E	S4
		Bargo River, Tahmoor, downstream	4°14′58.47″ S	150°36′25.37″ E	S5

**Table 2:** Correlations of the current results with different water quality parameters and indices, published by Ali et al. [1], for the same locations.

Parameters	DO%	pH	NO_2_	Turbidity	Total Dissolved Solids	Conductivity	Water Quality Index (WQI)	Contamination index (C_d_)	Heavy metal Evaluation index	Heavy metal Potential index (HPI)	Environmental Water Quality Index	Chlorophyll A µg/m^2^	Invert abundance	Invert richness	Invert density	SIGNAL 2 index	Average Sensitivity Grade
DO%	1	−0.786	−0.706	−0.446	−0.844	−0.841	0.299	−0.105	−0.776	−0.442	0.970 *	0.062	−0.534	−0.923	−0.162	−0.854	−0.904
pH	−0.786	1	0.978 *	0.765	0.925	0.897	−0.585	−0.100	0.806	0.827	−0.853	0.565	0.542	0.963 *	0.187	0.363	0.624
NO_2_	−0.706	0.978 *	1	0.881	0.945	0.924	−0.737	0.042	0.863	0.762	−0.750	0.617	0.66	0.912	0.357	0.29	0.47
Turbidity	−0.446	0.765	0.881	1	0.856	0.859	−0.968 *	0.412	0.882	0.448	−0.416	0.574	0.858	0.66	0.72	0.141	0.07
Total dissolved solids	−0.844	0.925	0.945	0.856	1	0.998 **	−0.745	0.278	0.969 *	0.551	−0.816	0.332	0.806	0.936	0.502	0.562	0.57
Conductivity	−0.841	0.897	0.924	0.859	0.998 **	1	−0.762	0.34	0.983 *	0.494	−0.797	0.286	0.84	0.916	0.55	0.588	0.551
Water Quality Index (WQI)	0.299	−0.585	−0.737	−0.968 *	−0.745	−0.762	1	−0.594	−0.830	−0.223	0.223	−0.461	−0.903	−0.480	−0.858	−0.094	0.12
Contamination index (Cd)	−0.105	−0.100	0.042	0.412	0.278	0.34	−0.594	1	0.505	−0.612	0.126	−0.401	0.778	−0.032	0.921	0.385	−0.249
Heavy metal evaluation index (HEI)	−0.776	0.806	0.863	0.882	0.969 *	0.983 *	−0.830	0.505	1	0.343	−0.693	0.208	0.925	0.829	0.691	0.59	0.437
Heavy metal potential index (HPI)	−0.442	0.827	0.762	0.448	0.551	0.494	−0.223	−0.612	0.343	1	−0.630	0.789	0.018	0.713	−0.297	−0.078	0.475
Environmental Water Quality Index	0.970 *	−0.853	−0.750	−0.416	−0.816	−0.797	0.223	0.126	−0.693	−0.630	1	−0.106	−0.387	−0.955 *	0.02	−0.725	−0.935
Chlorophyll A µg/m^2^	0.062	0.565	0.617	0.574	0.332	0.286	−0.461	−0.401	0.208	0.789	−0.106	1	0.075	0.326	−0.018	−0.563	−0.151
Invert abundance	−0.534	0.542	0.66	0.858	0.806	0.84	−0.903	0.778	0.925	0.018	−0.387	0.075	1	0.555	0.913	0.486	0.122
Invert richness	−0.923	0.963 *	0.912	0.66	0.936	0.916	−0.480	−0.032	0.829	0.713	−0.955 *	0.326	0.555	1	0.17	0.594	0.787
Invert density	−0.162	0.187	0.357	0.72	0.502	0.55	−0.858	0.921	0.691	−0.297	0.02	−0.018	0.913	0.17	1	0.243	−0.265
SIGNAL-2 index	−0.854	0.363	0.29	0.141	0.562	0.588	−0.094	0.385	0.59	−0.078	−0.725	−0.563	0.486	0.594	0.243	1	0.777
Average sensitivity grade	−0.904	0.624	0.47	0.07	0.57	0.551	0.12	−0.249	0.437	0.475	−0.935	−0.151	0.122	0.787	−0.265	0.777	1

* Correlation is significant at the 0.05 level (two-tailed), ** Correlation is significant at the 0.01 level (two-tailed). SIGNAL—Stream Invertebrate Grade Number-Average Level.
